# Supplementary material for: Different Impacts of Heat-Killed and Viable Lactiplantibacillus plantarum TWK10 on Exercise Performance, Fatigue, Body Composition, and Gut Microbiota in Humans
Source: Microorganisms. 2022 Nov 3;10(11):2181. doi: 10.3390/microorganisms10112181 (PMC9692508; doi:10.3390/microorganisms10112181)
Supplement: Supplementary file 1 [file microorganisms-10-02181-s001.zip › microorganisms-1971066-supplementary.pdf]

Microorganisms Supplementary Materials for:

# Different impacts of heat-killed and viable *Lactiplantibacillus plantarum* TWK10 on exercise performance, fatigue, body composition, and gut microbiota in humans

**Chia-Chia Lee[1,†], Yi-Chu Liao[1,†], Mon-Chien Lee[2], Yi-Chen Cheng[1], Shiou-Yun Chiou[1], Jin-Seng Lin[1], Chi-Chang Huang[2,*], Koichi Watanabe[1,3,*]**

[1] Culture Collection & Research Institute, SYNBIO TECH INC., Kaohsiung, Taiwan.
[2] Graduate Institute of Sports Science, National Taiwan Sport University, Taoyuan, Taiwan.
[3] Department of Animal Science and Technology, National Taiwan University, Taipei, Taiwan.

* Correspondence:
Chi-Chang Huang (john5523@ntsu.edu.tw)
Koichi Watanabe (koichi_wtnb@yahoo.co.jp)

† These authors contributed equally to this work and share first authorship

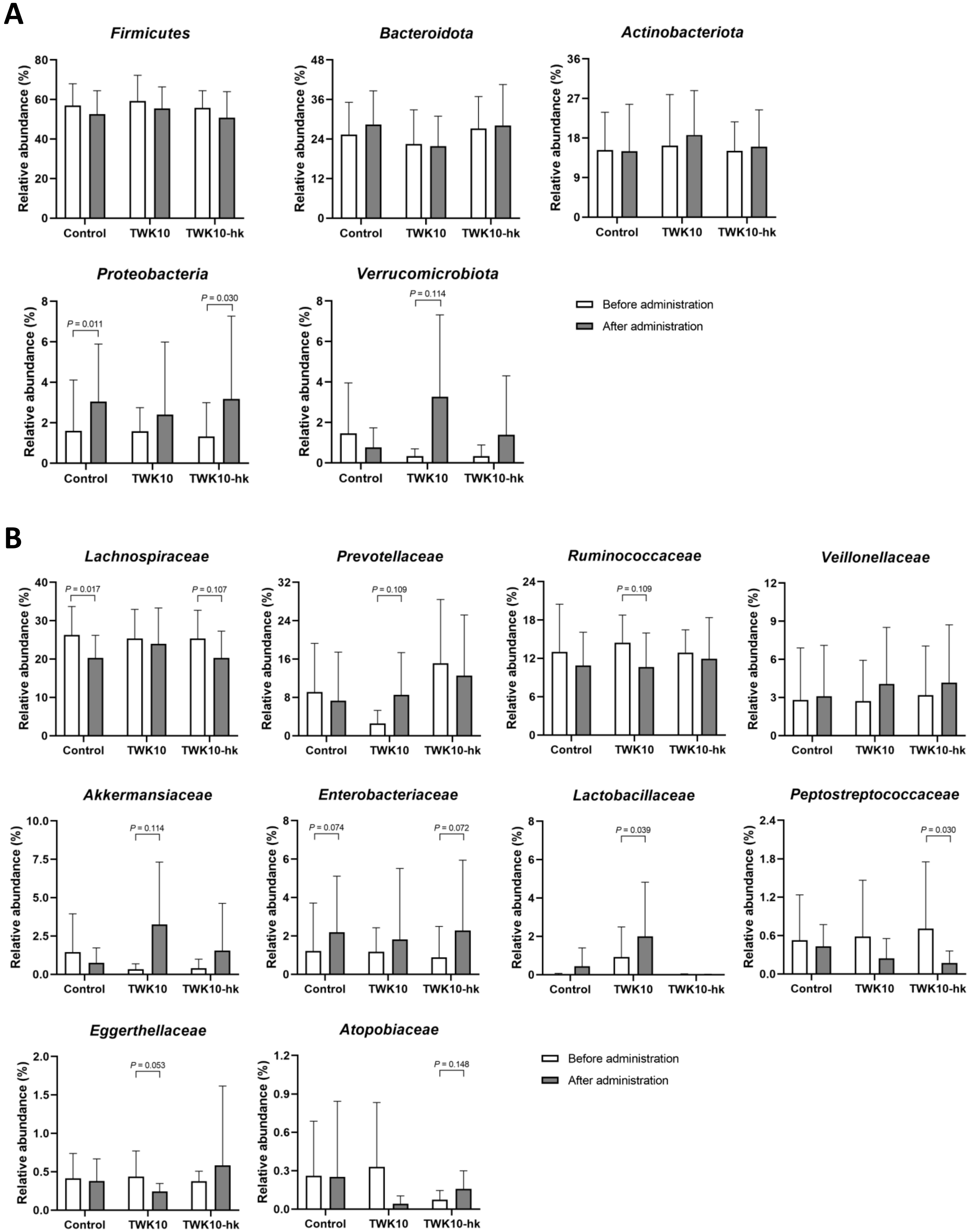


**Figure S1. Relative abundances of the major gut microbiota among groups.** Columns show the relative abundances of bacterial taxa at the (A) phylum and (B) family level in the Control, TWK10, and TWK10-hk groups compared with their baselines. Data shown as mean ± SD. Statistical differences between before and after administration were analyzed by Mann-Whitney *U* test.

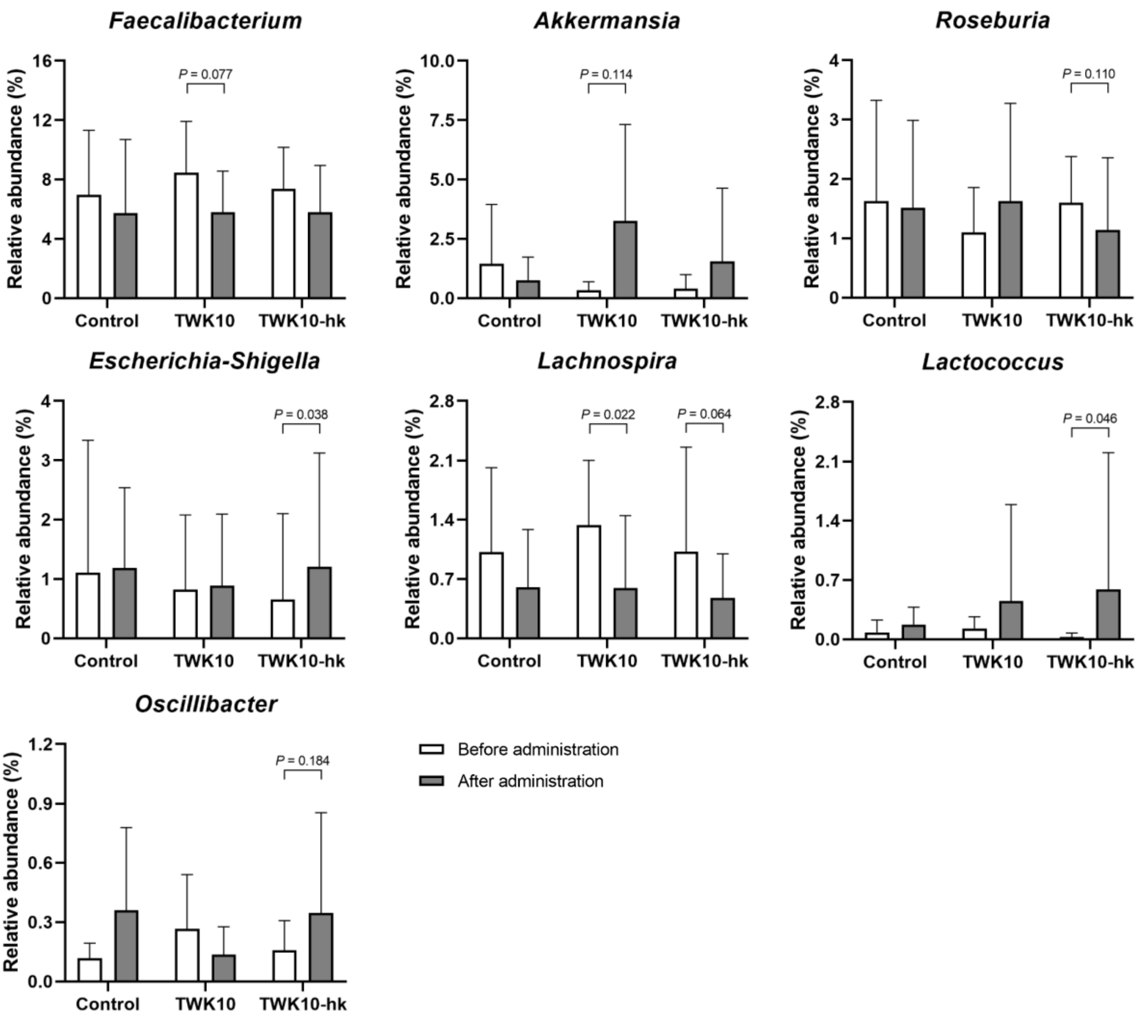


**Figure S2. Relative abundances of the major gut microbiota among groups.** Columns show the relative abundances of bacterial taxa at the genus level in the Control, TWK10, and TWK10-hk groups compared with their baselines. Data shown as mean ± SD. Statistical differences between before and after administration were analyzed by Mann-Whitney *U* test.
